# Life time use of illicit substances among adolescents and young people hospitalized in psychiatric hospital

**DOI:** 10.1038/s41598-023-28603-2

**Published:** 2023-02-01

**Authors:** Piotr Engelgardt, Maciej Krzyżanowski, Małgorzata Borkowska-Sztachańska, Agnieszka Wasilewska, Michał Ciucias

**Affiliations:** 1 Department of Pathomorphology and Forensic Medicine, University of Warmia and Mazury, Olsztyn, Poland; 2 Department of Psychiatry, University of Warmia and Mazury, Olsztyn, Poland; 3 Department of Anatomy, University of Warmia and Mazury, Olsztyn, Poland

**Keywords:** Health care, Medical research, Risk factors

## Abstract

Adolescents are known to be particularly vulnerable, compared to children and adults, to initiation of substance use and progression to problematic use. This study aimed to examine the prevalence and type of illicit drug use in a population of adolescents and young adults who were hospitalized in a psychiatric hospital. The purpose of the study was also to find the link between age, sex, type of admission and particular mental disorders and using psychoactive substances at least once in a lifetime. A 12-month retrospective cross-sectional analysis of medical records compiled for adolescent and youth psychiatric patients who had been admitted to the Regional Psychiatric Hospital in Olsztyn, Poland, between October 1, 2018, and September 30, 2019, was conducted. After analyzing the available medical records, 506 cases were included and analyzed. Data for the study were collected in an Excel spreadsheet from discharge reports, including data from psychiatric examinations, especially anamnesis. Subsequently, statistical calculations were performed. Lifetime prevalence of any illicit substance use (34.0%) was common. The most frequently used drug was Cannabis (29.2%), the next New Psychoactive Substance—NPS (14.2%) and Amphetamine (13.0%). The higher number of people declaring to take illicit substances was proportional to the increasing age. Except for the group 10–15 years, the subject group was dominated by males. The highest, statistically significant percentage of patients who declared taking illicit substances in general, was found in people with diagnoses F20–F29 (schizophrenia, schizotypal and delusional disorders) (55%), additionally, we found a statistically significant association between NPS use and these diagnoses. Only in the group of patients diagnosed with eating disorders no one declared taking psychoactive substances. However, the correlation between taking illicit drugs and the subgroups with diagnosed psychiatric diseases should be treated with caution because of the small sample size in some cases. Our findings have shown the significant prevalence of the phenomenon in this population. These data highlight the need to explore this population at high risk carefully.

## Introduction

Adolescence represents a developmental period that appears to be essential regarding substance use initiation and the development of mental and behavioural disorders due to psychoactive substance use^1,2^. The brain undergoes significant neurodevelopment between childhood and young adulthood. The maturation continues until around age 25. Adolescents are known to be particularly vulnerable, compared to children and adults, to initiation of substance use and progression to problematic use^3^. In epidemiological studies on illegal drug use among European teenagers and young adults, the prevalence of psychoactive substance use was found to be high—17% of adolescents reported using illicit drugs at least once^4,5^. In Poland, 22% of teenagers declared taking illicit substances at least once in their lifetime^5^. Cannabis was the most frequently used illicit drug by adolescents^4,5^, representing a constant prevalence of 16% in Europe since 2015; and adequately 21% in Poland. Use of other illicit drugs or other psychoactive substances (Inhalants, NPS, Pharmaceuticals) was less common among adolescents, with a prevalence of 0.7–9.2%. In Poland, the prevalence varies from 0.9 to 18%, depending on the substance type^5^. Scientific reports show that the chance of substance use and misuse increased with age^5,6^. The research has also shown that generally, males tend to have higher rates of substance use than females^6–8^. Further research has demonstrated that adolescent substance use increased the risk of school failure^2^ and suicide attempts^2,8^. In addition, substance use adolescents were more likely to be involved in serious risk behaviour, like criminal activities, fights, or drunk driving^1,2^. Several studies have assessed the significant link between substance use and psychopathology^9,10^. This kind of analogy is stressed both between adults and adolescents. People whose mental or behavioural disorders were the consequence of psychoactive substances tend to present the symptoms of their illness much more frequently. However, the very presence of mental diseases was linked to increased frequency of using psychoactive substances^8^. Compared with adolescents in the general population, adolescents hospitalized for mental illnesses had a higher prevalence of alcohol, nicotine and illicit drug consumption^8^.

Young people who suffered from mental diseases were substantially more prone to use illicit drugs. It is worth stressing the visible differences between the use of illicit substances by people representing different mental disorders. For those with Attention-deficit/hyperactivity disorder (ADHD), personality disorder, conduct disorder and affective disorders, the misuse and abuse of illicit substances were significantly higher^11,12^. On the opposite, the adolescent representing restrictive eating disorders was less prone to abuse alcohol, nicotine or drugs compared to other groups^13,14^.

There are also many reports in the literature on the links between psychoses and the use of psychoactive substances. In addition to the risk of inducing acute psychosis, regular use of several recreational drugs, especially amphetamine, methamphetamine and cannabis, is associated with the subsequent development of chronic psychosis or schizophrenia^15–17^.

One case series describing psychosis associated with acute recreational drug toxicity found cannabinoids and tryptamines to be the most frequently used substances^18^. In the group of adolescents and young adults with the first episode of psychosis, co-occurring use of psychoactive substances was reported in 74% of cases^19^. This co-occurrence is associated with less effective responses to treatment, reduced adherence to medical recommendations and, as a consequence, worsening of the course of the disease^20^. According to Martinotti et al., the use of NPS is common among young people/young adults and often among psychiatric patients who report psychotic symptoms at the onset of the disease. There is also significant evidence that NPS are a major risk factor for violence and aggression in patients suffering from serious mental disorders, as well as psychosis^21,22^. The correlation between psychoses and the use of psychoactive substances observed by many researchers has led to the identification of SIP (Substance Induced Disorder). Diagnoses of SIP were included in both the International Classification of Diseases-10 (ICD-10) and the Diagnostic and Statistical Manual of Mental Disorders—DSM-5^23,24^. In practical terms, many cases of SIP are difficult to distinguish clinically from PPD (Primary Psychiatric Disorder)^21^. Currently, some researchers, like Martinotti et al. aim to distinguish a novel clinical entity: Substance Related Exogenous Psychosis (SREP). They concluded that potent and highly rewarding NPS use is frequently associated with SREP^21^.

The current study aimed to investigate the prevalence and type of illicit drug use in a population of adolescents and young adults who were hospitalized in a psychiatric hospital. The purpose of the study was also to find the link between factors such as: age, sex and the type of admission (emergency/elective) and using illicit substances. Based on the previous research, we expected that the relationship between age and the prevalence of psychoactive substance consumption would be increasingly proportional. Another goal of this study was to examine the relationship between the use of psychoactive substances at least once in a lifetime and particular groups of psychiatric disorders in patients.

## Materials and methods

### Study design and data collection

The study was designed as a retrospective cross-sectional review of medical records written for adolescent and youth psychiatric (10–24 years old) patients admitted to the Regional Psychiatric Hospital in Olsztyn, Poland, between October 1, 2018, and September 30, 2019, were identified from the hospital medical database. All the medical documentation of patients was analyzed, including medical records, discharge summaries and consultation notes. Such data were collected during clerking of patients and routine consultations; thus, reporting of these outcomes depended on clinical inquiry and patient self-reporting. All data were recorded in an Excel spreadsheet. The essential data about patients was presented in the documentation included age, sex, psychiatric diagnosis according to ICD-10, the form of admission to the hospital (emergency-elective), taking illicit drugs (at least once in a lifetime) and the type of used substances. The substances that fall into the category of illicit drugs were all those that were used for intoxication or any other that affect the mind. That is why benzodiazepines and tramadol, which is classified as opioid, are mentioned in the study. On the other hand, the data concerning the use of alcohol and cigarettes was not taken into account.

The division was made based on education stages in Poland:

- primary school, gymnasium (< 15 years),
- high school (16–19 years),
- superior studies (20–24 years).

The study group was divided according to age criteria. The next step was to find the relationship between the age, sex, mental disorders, diseases, type of admission (emergency/elective) and declarations on taking psychoactive substances (all kinds of drugs and the three most popular of them). At this point, it is worth adding that emergency admissions included patients presenting symptoms of mental disorders that pose a threat to their lives; however, their somatic state was stable.

Patients with acute life-threatening symptoms of intoxication were initially treated in the Emergency Ward and then transferred to appropriate departments depending on their specific health problem and overall condition (Intensive Care Unit, Toxicology Department or others).

### Data analysis

All data was transferred into an IBM SPSS Statistical Package 27 (IBM Corp., Armonk, NY) and coded. Significance for all tests was initially determined at the level of *p* < 0.05, and adjusted for multiple testing with Bonferroni correction; three tests were conducted (Pearson chi-square, bivariate logistic regression, and multivariable logistic regression). Thus, the initial significance threshold was divided by the number of conducted tests, resulting in an adjusted significance threshold of *p* < 0.017. Individual cases were identified for incomplete medical reports and excluded from subsequent analyses. The remaining cases were included in the study. Data were expressed as frequencies, but percentage values were also provided. Since all of the analyzed data were categorical (e.g., sociodemographic factors, substance use, admission type, ICD diagnosis), Pearson chi-squared with Yates’s correction for continuity tests were conducted to determine the differences in the distribution of frequencies among the groups being compared (e.g., sex or age group).

In the next step, bivariate logistic regression was performed for any of the predictors separately, to assess their potential relationship with the illicit substances use (list of predictors in Table 5—results of the bivariate logistic regression). Subsequently, predictors that demonstrated a significant contribution to the regression model based on the omnibus tests of model coefficients were entered simultaneously into a multivariable logistic regression model, for the final examination of the correlations with the illicit substances use. A separate analysis in terms of running bivariate logistic regression and multivariable logistic regression was also conducted for the use of the Cannabis, AMP and NPS, separately. In the logistic regression, adjusted odds ratio, confidence interval and p values were presented.

### Human ethics and declarations sections, including consent to participate

The author declare that the study complies with the ethical guidelines and applicable local law. This study was approved by the local Bioethics Committee of the University of Warmia and Mazury in Olsztyn—Decision No. 24/2016 on June 30, 2016. The study was designed as a retrospective data analysis. Data from medical records were first blinded and then analyzed anonymously. Therefore informed consent, in this case, was not required.

## Results

### Demographic characteristics of patients

During the study period, the whole number of patients admitted to the hospital was 3506, 641 of whom were aged 10–24 years. 137 patients were excluded from the study group due to incomplete medical records. The remaining 506 cases were included in the study. Table 1 presents demographic data and the type of hospital admission of the study sample.

**Table 1:** Demographic data.

	N	%
Sex		
Female	260	51.4
Male	246	48.6
Age		
10	11	2.2
11	19	3.8
12	21	4.2
13	40	7.9
14	78	15.4
15	49	9.7
16	68	13.4
17	67	13.2
18	25	4.9
19	19	3.8
20	16	3.2
21	22	4.3
22	21	4.2
23	26	5.1
24	24	4.7
Age group		
10–15 years	218	43.1
16–19 years	179	35.4
20–24 years	109	21.5
Type of hospital admission		
Emergency	313	61.9
Elective	193	38.1

In the examined group there were no significant differences in the sex distribution (χ^2^ = 0.387; p = 0.534). The median age was 16 years, and the most numerous group were 14-year-olds, next 16 and 17-year-olds, while the least numerous were 10-year-olds. The examined group was divided according to age, taking into account the educational stage.

### Prevalence and types of illicit substances declared in the examined population

506 cases were analyzed in this study. 172 people (34%) from this group declared taking illicit drugs at least once in their lifetime, including 108 males and 64 females. 75 people declared having taken only one illicit substance (which stands for 43.6% of people declaring taking psychoactive substances in general), while 97 people declared taking more than one illicit substance (56.4% of people declaring taking illicit substances in general). There were no statistically significant differences in terms of the size of the groups of mono-drug users and polydrug users (χ^2^ = 3.094, p = 0.07). Table 2 presents the types of illicit drugs which were used by people according to the available medical data.

**Table 2:** The types of illicit drugs which were used by people according to the available medical data.

	N (male/female)	% of ID positive	% of total
Cannabis	148 (100/48)	86.0	29.2
NPS	72 (47/25)	41.8	14.2
AMP	66 (40/26)	38.4	13
BDZ	19 (13/6)	11.0	3.7
OPI	17 (10/7)	9.9	3.3
MDMA	16 (12/4)	9.3	3.1
COC	5 (5/0)	2.9	0.9
Others	5 (2/3)	2.9	0.9
MET	4 (2/2)	2.3	0.8
LSD	3 (2/2)	1.7	0.6

### The pattern of taking illicit drugs in age groups representing different sexes

This part of the paper provides the analysis concerning the number of people who declared using illicit drugs (any type, Cannabis, AMP, NPS) in particular age groups. Due to the low numbers in groups declaring taking other illicit substances, their analysis was not performed. The sex parameter was also taken into account in the study. Table 3 presents taking illicit substances according to age and sex with results of Pearson chi-squared tests.

**Table 3:** Taking illicit substances according to age and sex.

	Total (N = 506)	Any type (N = 172)	Cannabis (N = 148)	AMP (N = 66)	NPS (N = 72)
Age group	N (%)	ID + N (%)	N (%)	N (%)	N (%)
	Male N (%)	Male ID + N (%)	Male N (%)	Male N (%)	Male N (%)
	Female N (%)	Female ID + N (%)	Female N (%)	Female N (%)	Female N (%)
10–15	218 (43.1%)	33 (15.1%)	28 (18.9%)	8 (12.1%)	9 (12.5%)
	78 (15.4%)	9 (4.1%)	9 (6.08%)	1 (1.52%)	1 (1.39%)
	140 (27.7%)	24 (11%)	19 (12.84%)	7 (10.61%)	8 (11.11%)
16–19	179 (35.4%)	72 (40.2%)	63 (42.6%)	29 (43.9%)	33 (45.8%)
	84 (16.6%)	43 (24.0%)	41 (27.70%)	16 (24.24%)	21 (29.17%)
	95 (18.8%)	29 (16%)	22 (14.86%)	13 (19.70%)	12 (16.67%)
20–24	109 (21.5%)	67 (61.5%)	57 (38.5%)	29 (43.9%)	30 (41.7%)
	84 (16.6%)	56 (51.4%)	50 (33.78%)	23 (34.85%)	25 (34.72%)
	25 (4.9%)	11 (10%)	7 (4.73%)	6 (9.09%)	5 (6.94%)
Results of Pearson chi-squared tests (p < 0.05)					
10–15 vs 16–19		χ^2^ = 34.8 **p < 0.001**	χ^2^ = 26.89 **p < 0.001**	χ^2^ = 16.91 **p < 0.001**	χ^2^ = 19.97 **p < 0.001**
16–19 vs 20–24		χ^2^ = 11.4 **p < 0.001**	χ^2^ = 7.25 **p = 0.007**	χ^2^ = 3.84 p = 0.05	χ^2^ = 2.68 p = 0.10
Sex overall		χ^2^ = 20.106 **p < 0.001**	χ^2^ = 29.35 **p < 0.001**	χ^2^ = 3.90 p = 0.048	χ^2^ = 8.68 **p = 0.003**
Sex, age group 10–15		χ^2^ = 0.827 p = 0.36	χ^2^ = 0.05 p = 0.827	χ^2^ = 1.05 p = 0.306	χ^2^ = 1.49 p = 0.222
Sex, age group 16–19		χ^2^ = 7.081 **p = 0.008**	χ^2^ = 12.21 **p < 0.001**	χ^2^ = 0.65 p = 0.421	χ^2^ = 3.91 p = 0.048
Sex, age group 20–24		χ^2^ = 3.277 p = 0.07	χ^2^ = 6,46 **p = 0.011**	χ^2^ = 0.01 p = 0.938	χ^2^ = 0.49 p = 0.481

Significant values are in bold.

In the group of 10–15 year-olds none of the children under the age of 13 declared taking illicit drugs. In fact, all the cases of drug-taking can be included in the group of 13–15-year-olds. These are precisely 33 cases among 167 children aged 13–15. At this point, it is worth paying attention to the sex disproportion in such groups. In the group of 10–15 year-olds girls were prevailing over boys (64.2% vs 35.8%). However, in the rest of the groups, the male sex was prevailing over the female one (16–19 years—22.9% vs 77.1%; 20–24 years—33.3% vs 66.7%).

These statistics show that in the groups without the age division, as well as in the groups 16–19 and 20–24, the majority of people declaring taking illicit drugs are men. The age group 10–15 serves as an exception. In this particular group, there were no differences between the number of girls and boys declaring taking illicit drugs. In order to verify the results of the obtained Pearson chi-squared tests, logistic regressions will be carried out in the further part of the work.

### The pattern of taking illicit drugs according to the admission type (emergency/elective)

There was an additional analysis of the correlation between the declared use of illicit drugs and the admission type (emergency/elective). Among those declaring taking illicit drugs, 115 cases (66.9% of the subgroup) were emergency admissions. On the other hand, for people who denied taking illicit drugs at any time in their lifetime, 198 cases (59.0% of this subgroup) were emergency admissions, while 136 cases (40.7%) were elicit admissions. Thus, the percentage of people admitted in the emergency mode and having declared taking illicit drugs, was higher than in the group of people denying taking such substances. Nonetheless, the statistical calculations showed no statistically significant differences in the admission type and taking illicit substances declared by people (see Table 4).

**Table 4:** Taking illicit substances according to the admission type.

	Total (N = 506)	Any type (172)	Cannabis (N = 148)	AMP (N = 66)	NPS (N = 72)
Emergency admission	313	N = 115	N = 98	N = 43	N = 41
Results chi-squared tests		χ^2^ = 2.452 p = 0.117	χ^2^ = 1.35 p = 0.245	χ^2^ = 0.19 p = 0.665	χ^2^ = 0.67 p = 0.412

### Comparison between use of illicit drugs and psychiatric diagnoses

Subsequently, types of psychiatric diseases or disorders according to ICD-10 diagnosed in a particular group were checked against the declared drug use. The precise data is presented in Table 5.

**Table 5:** Use illicit drugs (ID) according to the psychiatric diagnosis made during the hospitalization.

Diagnoses—ICD10 groups*	Total (N = 506)		Any type + (N = 172)		Most popular	Cannabis +		AMP + (N = 66)		NPS + (N = 72)	
	N	%	ID + N	%		N	%	N	%	N	%
	Male	Male	Male	Male		Male	Male	Male	Male	Male	%
	Female	Female	Female	Female		Female	Female	Female	Female	Female	%
F00–F09 Organic, including symptomatic, mental disorders	9	1.80	6	66.70	Cannabis	4	2.70	0	0	1	1.40
	*7*	1.40	5	55.60		3	2.03	0	0	1	1.39
	*2*	0.40	1	11		1	0.68	0	0	0	0
F10–F19 Mental and behavioral disorders due to psychoactive substance use	57	11.30	50	87.70	Cannabis	45	30.40	27	40.90	27	37.50
	46	9.10	40	70.20		38	25.68	22	33.33	21	29.17
	11	2.20	10	18		7	4.73	5	7.58	6	8.33
F11–F19 Mental and behavioral disorders due to psychoactive substance use (alcohol excluded)	43	8.50	43	100.00	Cannabis	38	25.70	25	37.90	24	33.30
	35	6.90	35	81.40		33	22.30	20	30.30	19	26.39
	8	1.60	8	19		5	3.38	5	7.58	5	6.94
F20–F29 Schizophrenia, schizotypal and delusional disorders	40	7.90	22	55.00	Cannabis, NPS	18	12.20	10	15.20	18	25.00
	26	5.10	16	40.00		16	10.81	4	6.06	12	16.67
	14	2.80	6	− 15		2	1.35	6	9.09	6	8.33
F30–F39 Mood [affective] disorders	44	8.70	11	25.00	Cannabis	10	6.80	2	3.00	2	2.80
	19	3.80	8	18.20		7	4.73	2	3.03	2	2.78
	25	4.90	3	7		3	2.03	0	0	0	0
F40–F48 Neurotic, stress-related and somatoform disorders	34	6.70	15	44.10	Cannabis	14	9.50	5	7.60	4	5.60
	9	1.80	6	17.60		6	4.05	1	1.52	2	2.78
	25	4.90	9	26		8	5.41	4	6.06	2	2.78
F50–F59 Behavioral syndromes associated with physiological disturbances and physical factors	6	1.20	0	0	–	0	0	0	0	0	0
	0	0	0	0		0	0	0	0	0	0
	6	1.20	0	0		0	0	0	0	0	0
F60–F69 Disorders of adult personality and behavior	6	1.20	3	50.00	Cannabis, NPS, AMP	2	1.40	2	3.00	2	2.80
	5	1.00	3	50.00		2	1.35	2	3.03	2	2.78
	1	0.20	0	0		0	0	0	0	0	0
F70–F79 Mental retardation	71	14.00	9	12.70	Cannabis, NPS	3	2.00	5	7.60	8	11.10
	45	8.90	3	4.20		2	1.35	0	0	2	2.78
	26	5.10	6	− 8		1	0.68	5	7.58	6	8.33
F80–F89 Disorders of psychological development	42	8.30	3	7.10	Cannabis	1	0.70	1	1.50	2	2.80
	30	5.90	3	7.10		1	0.68	1	1.52	2	2.78
	12	2.40	0	0		0	0	0	0	0	0.00
F90–F99 Behavioral and emotional disorders with onset usually occurring in childhood and adolescence	79	15.60	22	27.80	Cannabis	19	12.80	8	12.10	6	8.30
	32	6.30	9	11.40		9	6.08	4	6.06	3	4.17
	47	9.30	13	16		10	6.76	4	6.06	3	4.17
Z03 Medical observation and evaluation for suspected diseases and conditions, ruled out	218	43.10	71	32.60	Cannabis	65	43.90	24	36.40	24	33.30
	88	17.40	42	19.30		40	27.03	16	24.24%	15	20.83
	130	25.70	29	13		25	16.8	8	12.12	9	12.50

Results of Pearson chi-squared tests (p < 0.05)											
			Any type+			Cannabis+		AMP+		NPS+	
F00–F09 Organic, including symptomatic, mental disorders			χ^2^ = 3.00			χ^2^ = 0.41		χ^2^ = 0.45		χ^2^ = 0	
			p = 0.083			p = 0.524		p = 0.5		p = 1	
F10–F19 Mental and behavioral disorders due to psychoactive substance use			χ^2^ = 79.96			χ^2^ = 73.74		χ^2^ = 63.17		χ^2^ = 54.61	
			**p < 0.001**			**p < 0.001**		**p < 0.001**		**p < 0.001**	
F11–F19 Mental and behavioral disorders due to psychoactive substance use (alcohol excluded)			χ^2^ = 88.07			χ^2^ = 76.06		χ^2^ = 79.75		χ^2^ = 62.73	
			**p < 0.001**			**p < 0.001**		**p < 0.001**		**p < 0.001**	
F20–F29 Schizophrenia, schizotypal and delusional disorders			χ^2^ = 7.56			χ^2^ = 4.94		χ^2^ = 4.74		χ^2^ = 32.40	
			**p = 0.006**			p = 0.026		p = 0.029		**p < 0.001**	
F30–F39 Mood [affective] disorders			χ^2^ = 1.32			χ^2^ = 0.689		χ^2^ = 2.31		χ^2^ = 2.90	
			p = 0.250			p = 0.406		p = 0.128		p = 0.089	
F40–F48 Neurotic, stress-related and somatoform disorders			χ^2^ = 1.28			χ^2^ = 1.90		χ^2^ = 0.001		χ^2^ = 0.031	
			p = 0.270			p = 0.168		p = 0.976		p = 0.860	
F50–F59 Behavioral syndromes associated with physiological disturbances and physical factors			χ^2^ = 1.78			χ^2^ = 1.29		χ^2^ = 0.12		χ^2^ = 0.174	
			p = 0.182			p = 0.256		p = 0.729		p = 0.676	
F60–F69 Disorders of adult personality and behavior			χ^2^ = 0.16			χ^2^ = 0		χ^2^ = 0.76		χ^2^ = 0.573	
			p = 0.690			p = 1		p = 0.383		p = 0.449	
F70–F79 Mental retardation			χ^2^ = 16.72			χ^2^ = 23.17		χ^2^ = 1.94		χ^2^ = 0.297	
			**p < 0.001**			**p < 0.001**		p = 0.163		p = 0.586	
F80–F89 Disorders of psychological development			χ^2^ = 14.71			χ^2^ = 14.64		χ^2^ = 3.63		χ^2^ = 2.58	
			**p < 0.001**			**p < 0.001**		p = 0.056		p = 0.108	
F90–F99 Behavioral and emotional disorders with onset usually occurring in childhood and adolescence			χ^2^ = 1.57			χ^2^ = 0.97		χ^2^ = 0.44		χ^2^ = 2.78	
			p = 0.209			p = 0.326		p = 0.507		p = 0.095	
Z03 Medical observation and evaluation for suspected diseases and conditions, ruled out			χ^2^ = 0.24			χ^2^ = 0.02		χ^2^ = 1.13		χ^2^ = 2.86	
			p = 0.622			p = 0.904		p = 0.287		p = 0.091	

*The total number of all diagnoses is bigger than the total number of members from the examined group because of double and triple diagnoses.

Significant values are in bold and italics.

In 86 cases (17%) there were examples of double diagnoses, while in 7 cases (1.4%) there were 3 different comorbid mental diseases. The rest of the group members—413 patients were diagnosed with single diseases. Discussing the diagnoses of the F10–F19 group in detail led to the following conclusion: in 17 cases (3.4% of all cases) the diagnosis was: Alcohol-related disorders (F10). However, in 43 cases, the diagnosis included groups F11–F19. The total number of diagnoses (F10, and F11–F19) was equal to 60, while the number of diagnoses F10–F19 was equal to 57. Such a phenomenon can be explained based on three cases with a double diagnosis (F10 and F19 comorbidity). When it comes to F11–F19 groups, there was one case of F11 (Opioid-related disorders), 7 cases from F12 (Cannabis-related disorders), 1 case from F15 (Other stimulant-related disorders), and 34 cases from the F19 group (Other psychoactive substance-related disorders). In groups F00–F09 (Mental disorders due to known physiological conditions), eight cases were diagnosed with F07—Personality and behavioural disorders due to known physiological conditions. In groups F50–F59 all 6 diagnoses proved F50 (eating disorders), including 5 anorexia nervosa (F50.0) and one bulimia nervosa (F50.2).

### Results of further statistical analysis (bivariate and multivariable regression)

In order to check the correctness of the conclusions obtained by the Pearson chi-squared test on the relationship between substance use and sex, age and drug-related disorders the logistic regression (bivariate and multivariable) was used.

Bivariate logistic regression results demonstrated a significant association between illicit substance use and factors such as: sex, age (significant association found for age overall, 10–15, and 20–24 age groups, but not for 16–19 age group) and diagnoses: F10–F19, F20–F29, F70–F79, F80–F89 (see Table 6).

**Table 6:** Results of bivariate (single) logistic regression.

Variables	Any type (172/506)	Cannabis (148/506)	AMP (66/506)	NPS (72/506)
	AOR (adjusted odds ratio) CI 95% (confidence interval 95%) *p* value	AOR CI 95% *p*	AOR CI 95% *p*	AOR CI 95% *p*
Sex				
Sex: male	2.40 (1.64–3.50) **p < 0.001**	3.03 (2.01–4.56) **p < 0.001**	1.75 (1.03–2.98) p = 0.037	2.23 (1.32–3.75) **p = 0.003**
Age group				
Age overall	1.29 (1.22–1.37) **p < 0.001**	1.24 (1.18–1.31) **p < 0.001**	1.24 (1.16–1.34) **p < 0.001**	1.23 (1.14–1.31) **p < 0.001**
Age group: 10–15	0.19 (0.12–0.30) **p < 0.001**	0.21 (0.13–0.32) **p < 0.001**	0.15 (0.07–0.32) **p < 0.001**	0.15 (0.07–0.31) **p < 0.001**
Age group 16–19	1.52 (1.04–2.23) p = 0.029	1.56 (1.05–2.31) p = 0.027	1.52 (0.90–2.56) p = 0.115	1.68 (1.01–2.78) p = 0.044
Age group 20–24	4.43 (2.84–6.93) **p < 0.001**	3.67 (2.36–5.72) **p < 0.001**	3.51 (2.04–6.05) **p < 0.001**	3.20 (1.88–5.43) **p < 0.001**
Diagnoses				
F00–F09 Organic, including symptomatic, mental disorders	3.99 (0.98–16.14) p = 0.053	1.96 (0.52–7.41) p = 0.321	p = 0.999	0.74 (0.09–6.074) p = 0.786
F10–F19 Mental and behavioral disorders due to psychoactive substance use	19.14 (8.45–43.37) **p < 0.001**	12.56 (6.40–24-63) **p < 0.001**	9.44 (5.10–17.49) **p < 0.001**	8.06 (4.40–14.75) **p < 0.001**
F20–F29 Schizophrenia, schizotypal and delusional disorders	2.58 (1.34–4.95) **p = 0.005**	2.21 (1.14–4.30) p = 0.19	2.52 (1.16–5.45) p = 0.023	6.54 (3.27–13.04) **p < 0.001**
F30–F39 Mood [affective] disorders	0.62 (0.31–1.27) p = 0.190	0.69 (0.33–1.43) p = 0.318	0.29 (0.07–1.25) p = 0.098	0.26 (0.06–1.12) p = 0.072
F40–F48 Neurotic, stress-related and somatoform disorders	1.54 (0.78–3.20) p = 0.200	1.76 (0.86–3.58) p = 0.117	1.15 (0.43–3.11) p = 0.77	0.79 (0.27–2.31) p = 0.668
F50–F59 Behavioral syndromes associated with physiological disturbances and physical factors	0 0.0 p = 0.999	p = 0.999	p = 0.999	p = 0.999
F60–F69 Disorders of adult personality and behavior	1.96 (0.39–9.81) p = 0.410	1.21 (0.22–6.67) p = 0.828	3.39 (0.61–18.93) p = 0.163	3.06 (0.55–17.05) p = 0.201
F70–F79 Mental retardation	0.24 (0.12–0.50) **p < 0.001**	0.09 (0.03–0.29) **p < 0.001**	0.47 (0.18–1.21) p = 0.121	0.75 (0.34–1.63) p = 0.467
F80–F89 Disorders of psychological development	0.13 (0.04–0.44) **p < 0.001**	0.52 (0.01–0.38) p = 0.04	0.15 (0.02–1.11) p = 0.063	0.28 (0.07–1.18) p = 0.084
F90–F99 Behavioral and emotional disorders with onset usually occurring in childhood and adolescence	0.71 (0.42–1.21) p = 0.211	0.73 (0.42–1.27) p = 0.265	0.71 (0.32–1.56) p = 0.400	0.45 (0.19–1.07) p = 0.072
Z03 Medical observation and evaluation for suspected diseases and conditions, ruled out	0.89 (0.62–1.30) p = 0.561	1.04 (0.71–1.54) p = 0.826	0.72 (0.42–1.23) p = 0.233	0.61 (0.36–1.04) p = 0.071
Admission type				
Emergency admission	0.72 (0.49–1.06) p = 0.097	0.77 (0.52–1.15) p = 0.207	0.85 (0.49–1.47) p = 0.569	1.277 (0.077–2.19) p = 0.343

Significant values are in bold.

Regarding the specific substances use, in the bivariate regression model age overall, age groups: 10–15, 20–24 and F10–F19 diagnosis showed significant association with all subgroups (Any type, Cannabis, AMP, NPS). Sex showed a significant association with all groups except AMP. Whereas F20–F29 diagnoses were correlated significantly with Any type and NPS, but not Cannabis and AMP use. Both F70–F79 and F80–F89 diagnoses were linked directly with the use of the Any type and F70–F79 with Cannabis. The detailed results of the analysis are presented in Table 6.

Further examination with the multivariable logistic regression resulted in finding the significant relationship between the illicit substance use and: sex, age, and presence of diagnoses: F10–F19 and F70–F79 and F80–F89. The significant associations with sex, age, and diagnoses: F10–F19 and F70–F79 were found in the case of the use of all illicit drugs. The subgroup Cannabis was closely correlated with sex, age, and diagnoses: F10–F19, F70–F79 and F80–F89. NPS use correlated significantly with age, F10–F19, and F20–F29 diagnoses, whereas only the age and F10–F19 diagnoses were significantly associated with AMP use. The unusual analysis results in the F50–F69 were most probably caused by the small number of cases in this F50–F59 group (N = 6) along with no reports of illicit substance use there. Dichotomous age groups variables were found to be collinear, thus they were excluded from the multivariable logistic regression model. Instead, a continuous overall age variable was entered into the regression model. Concerning the single/many illicit substance usage across the age groups among the specific substance (AMP, NPS and cannabis) users, the logistic regression (both bivariate, and multivariable) were not conducted due to possible high separation between age group and single/poly drug use variable (p value ranged from 0.99 to 1). The detailed results are presented in Table 7.

**Table 7:** Results of multivariable logistic regression (variables were entered simultaneously into the regression model). Only variables contributing significantly to the model were entered.

Variables	Any type (172/506)	Cannabis (148/506)	AMP (66/506)	NPS (72/506)
	AOR (adjusted odds ratio) CI 95% (Confidence Interval 95%) *p* value	AOR CI 95% *p*	AOR CI 95% *p*	AOR CI 95% *p*
Sex				
Sex: male	1.65 1.05–2.6 p = 0.030	2.595 (1.62–4.15) **p < 0.001**	0.925 (0.495–1.729) p = 0.806	1.29 (0.712–2.338) p = 0.401
Age				
Age overall	1.19 1.11–1.28 **p < 0.001**	1.14 (1.06–1.22) **p < 0.001**	1.163 (1.066–1.269) **p < 0.001**	1.111 (1.022–1.209) **p = 0.014**
Diagnoses				
F00–F09 Organic, including symptomatic, mental disorders	2.62 0.59–12.11 p = 0.217	1.419 (0.31–6.49) p = 0.652	NA	0.643 (0.074–5.553) p = 0.688
F10–F19 Mental and behavioral disorders due to psychoactive substance use	7.36 3.07–17.66 **p < 0.001**	4.718 (2.22–10.01) **p < 0.001**	4.717 (2.28–9.758) **p < 0.001**	3.846 (1.873–7.896) **p < 0.001**
F20–F29 Schizophrenia, schizotypal and delusional disorders	0.99 0.43–2.33 p = 0.998	0.791 (0.33–1.87) p = 0.594	0.961 (0.396–2.329) p = 0.929	3.37 (1.562–7.269) **p = 0.002**
F50–F59 Behavioral syndromes associated with physiological disturbances and physical factors	NA	NA	NA	NA
F60–F69 Disorders of adult personality and behavior	0.44 0.07–2.93 p = 0.396	0.304 (0.042–2.19) p = 0.237	2.067 (0.279–15.319) p = 0.478	2.021 (0.307–13.302) p = 0.464
F70–F79 Mental retardation	0.31 0.14–0.68 **p = 0.004**	0.104 (0.031–0.35) **p < 0.001**	0.895 (0.326–2.457) p = 0.83	1.134 (0.482–2.667) p = 0.773
F80–F89 Disorders of psychological development	0.23 0.07–0.79 p = 0.020	0.08 (0.01–0.61) **p = 0.015**	0.258 (0.033–2.018) p = 0.197	0.42 (0.094–1.881) p = 0.257
F90–F99 Behavioral and emotional disorders with onset usually occurring in childhood and adolescence	2.62 0.59–12.11 p = 0.217	1.419 (0.31–6.49) p = 0.652	NA	0.643 (0.074–5.553) p = 0.688
Z03 Medical observation and evaluation for suspected diseases and conditions, ruled out	7.36 3.07–17.66 p < 0.001	4.718 (2.22–10.01) p < 0.001	4.717 (2.28–9.758) p < 0.001	3.846 (1.873–7.896) p < 0.001

Significant values are in bold.

## Discussion

In the entire study group, 34% declared using illicit drugs throughout their lives, while in the group of adolescents (10–19 years old), it was 26% of people. The prevalence rate in this study was 9% higher than those typical for the European population. At the same time, it was slightly higher (4 percentage points) than the recent data given for the Polish population according to ESPAD, which was 22%^6^.

The results of this study were compared to the data from other studies covering similar groups of patients (adolescent inpatients of a psychiatric facility). In these studies, the use of illicit drugs was at the level of 36% in a group of 25 patients aged 12–17 years^25^, and at the level of 61% in a group of 70 patients aged 14–17^26^. Taking into account the differences concerning group sizes and age, such data is incomparable with the data collected in our research. Nevertheless, it is clear from both our data and the literature^8,11,25,26^ that, compared to the adolescent population in general, psychiatric patients in this age group are more likely to use prohibited substances. Thus, adolescent psychiatric patients require more attention due to the higher risk of developing addiction and the consequences that follow.

The most commonly used drugs in the European population are Cannabis, and then as follows Cocaine, Amphetamines (AMP) and 3,4-methylenedioxy-methamphetamine (MDMA)^4,5^. Patients from the examined group declared the use of cannabis as the most frequent (29.2% of the whole group), the second most popular substance were NPS (14.2% of the whole group), AMP (13% of the whole group), and then much less popular benzodiazepines (3.7% of the whole group), opioids and MDMA (a few per cent of cases). The use of Cocaine was declared by only 0.9% of examined patients. With the constantly growing access to cannabis products in society, it is necessary to educate about the risks associated with their use. Recreational use of cannabis by people sensitive to psychosis-inducing substances can lead to severe and lasting mental disorders in at-risk adolescents. Cannabis use is a highly-modifiable risk factor that can prevent PPDs from occurring in society. Medical staff is obliged to educate their patients, families and communities about the dangers of cannabis use^27,28^. The popularity of NPS in our group can be explained by the young age of the examined, as according to the literature these substances are especially popular among young people^22,29–33^. On the other hand, cocaine is relatively non-popular in Poland compared to other types of drugs^5,6^. It may be a consequence of its high price, compared to other illicit drugs in Poland.

Among the people who declared using illicit drugs in the past, the prevailing group was those who used more than one substance over the ones using only a single one (56.4% versus 43.6%). The following observations were similar to those that can be found in the literature^34^. This situation makes treatment and therapy difficult, both in acute poisoning and chronic admission. This is due to the atypical symptomatology, especially in the case of taking illegal substances, where it is not known what active substance is hidden under a random name. In addition, the interaction between multiple drugs can increase the neurological, physiological, and psychological impact on the user. Also, it could potentially increase the negative consequences of polydrug abuse^35^.

Based on the literature data, it can be admitted that in the adolescent population, the number of drug users increases with age^5,6^. The analysis presented in this paper leads to the same conclusions. There is a substantial difference between the youngest age group and the two others when it comes to the possibility of using psychoactive substances (in the subgroup with all kinds of illicit drugs as well as in the subgroups: Cannabis, AMP and NPS). The possibility of using them in a way that is statistically significant increases along with age. The following conclusion applies also to the particular substances that were analyzed (Cannabis, AMP, NPS).

Our research highlights the higher frequency of using illicit drugs of any type, NPS and Cannabis among young men, compared to women. However, such results were obtained in Pearson chi-squared test and in bivariate (single) logistic regression in the subgroup with all kinds of illicit drugs as well as in the subgroups: Cannabis and NPS. Those results were confirmed by the multivariable logistic regression only for Cannabis subgroups. What is more, while analyzing the frequency of taking illicit drugs according to sex, it was noticed that in the group aged 10–15, girls prevailed over boys (17.1% versus 11.5%), although this difference was not statistically significant (Pearson chi-squared tests). In the rest of the age subgroups that included children resembling the age above secondary school in Poland, the males were prevailing and the differences between sexes were statistically significant. The prevalence of girls in younger groups is in accordance with the information from other publications^10,36^. It may be well assumed that the following observations are rooted in the literature covering the differences in the maturation of girls and boys. It is, in general, believed that boys’ brains are developing slower than girls’, which may account for the slower biological and physical maturation of male teenagers^37,38^.

In the given study there was no correlation between the admission type (elective/emergency) and declarations of using illicit substances (Present in a subgroup with any type of illicit drugs as well as in subgroups: Cannabis, AMP and NPS). This result should be treated with caution.

In our opinion, it may result from the specificity of health care in Poland, because, as mentioned in the Methodology, patients with acute life-threatening symptoms of intoxication were initially treated in the Emergency Ward. Then they were transferred to appropriate departments, depending on their specific health problem and overall condition (Intensive Care Unit, Toxicology Department or others).

According to the prevalence of the use of illicit drugs in the lifetime in the subgroups diagnosed with other psychiatric diseases, the results presented in this paper partially differ in terms of frequency from the ones from the data collected in the literature covering the prevalence of using illicit drugs by people with different disorders.

In the subgroup with diagnosed eating disorders (F50–F59), none of the patients declared taking illicit substances during their lifetime. While other authors have reported that the use of illicit substances among adolescents with eating disorders is less common than among other psychiatric conditions in this group, they address the problem of the coexistence of eating disorders and psychoactive substance use, emphasizing its importance^13,14^. However, in our case, the small size of this subgroup means that these results are not statistically significant.

Data from the literature show the visible association between the use of illicit substances and schizophrenia spectrum disorders. A recent paper reported the relevance of these substance-related exogenous psychoses in adolescents and young adults, evidencing specific clinical presentations^21^. Our research also highlights the differences between the number of patients with schizophrenia spectrum disorders diagnosis who either declared or denied using illicit substances. The results of the Pearson chi-squared test were statistically significant for all analyzed subgroups. The bivariate regression model showed a significant association with all subgroups except Cannabis. Further examination with the multivariable logistic regression resulted in finding a significant relationship only in NPS subgroup. Such results correlate with the data provided by other authors in terms of an increased likelihood of developing psychoses among people using various illicit substances from NPS group^18,21,39,40^. According to Martinotti et al., the use of potent and highly rewarding NPS is frequently associated with SREP^21^. For example, synthetic cannabinoids, one of the subgroups of NPS, may accelerate the severity of the disorder by inducing psychotic relapse in patients with previously diagnosed mental disorders and those at high risk. This seems especially true for patients suffering from schizophrenia and substance-induced psychosis^41–43^. Papers on psychosis and synthetic cannabinoids suggest that they have stronger physiological and psychological effects than THC and can either aggravate previously stable psychotic symptoms or induce new psychosis^39^. Long-term psychosis and vulnerability to violence have been reported with the use of synthetic cathinones, another NPS group representative^40^. Although according to reports, SIP is associated with the development of severe mental illness, in larger pivotal studies, 24% to 32% of patients with SIP subsequently developed schizophrenia spectrum disorder or bipolar disorder^44^.

The methodological limitations of our work do not allow us to distinguish to what extent the diagnoses from the F20–F29 group in people declaring the use of psychoactive substances are actually PPD and to what extent they could be treated as consequences of taking psychoactive substances.

It was not possible to reach such a correlation with the literature data in the case of other illnesses. In the case of people from subgroups with illnesses from groups F70–F79: Mental retardation and F80–F89: Disorders of psychological development, the lesser likelihood of using illicit substances in general and Cannabis were observed. It was impossible to find statistically significant correlations in people who declared using AMP and NPS. The reason for that could be the smaller number of people with the diagnoses discussed who declared or denied the use of AMP and NPS. The authors were unable to find publications describing the problem of illicit drug use by adolescents diagnosed with mental retardation or psychological developmental disorders. Existing publications on substance use by the general population of people with intellectual disabilities emphasize the need for further study of this population in the area^45,46^. We suppose that the very nature of these conditions resulting in greater or lesser life awkwardness may imply a lower tendency to use illicit drugs by these adolescents.

People diagnosed from the group F10–F19: Mental and behavioural disorders due to psychoactive substance use did not undergo an analysis, as the specificity of these disorders makes this apparent.

In the case of other diagnoses, there were no statistically significant correlations. The statistically important association between the declared use of illicit substances and the appearance of diseases from the group F00–F09 has not been found. Even though the percentage of people declaring its use was the highest (67%). This fact can be explained by the small number of this group which included only 9 people.

The most numerous group in the examined population—the patients diagnosed with Z03 is heterogeneous, which in our opinion is the reason for the lack of dependence between people with such a diagnosis and declarations of taking medications. It is possible that within the time being, becoming mature and possible hospitalizations some of the cases will be provided with the proper diagnosis concerning the psychiatric disorders.

The strong point of the study is the relatively big and well-defined group (506 patients of the psychiatric hospital, hospitalized during the year).

The low number of some of the examined subgroups and the methodological differences, eg. different disease classifications (ICD-10, DSM-IV) could be the reason for the differences between the data presented in this paper and the information presented in the literature.

Another weak point of the study is the way these data were collected. It did not allow for a more precise assessment of, e.g. risk factors (serious risky behaviour, upbringing in an incomplete family, problems with learning etc.) or psychosocial consequences of using illicit drugs.

The retrospective analysis of medical documentation due to any patient’s highly individual psychiatric documentation did not give a chance for a very thorough study of separate factors.

## Conclusions and implications

Our findings have shown the significant prevalence of the phenomenon in population of adolescent and youth psychiatric patients in Poland. Our results indicate that the older the people within a certain group, the higher is the likelihood of using illicit substances. Additionally, we found a statistically significant association between NPS use and schizophrenia spectrum disorders (F20–F29). Nowadays, there is a visible need for further studies aimed at e.g., validating the risk factors and psychological consequences for the local population.

## Data Availability

The data used and analyzed during the research is available from the corresponding author upon reasonable request. All authors had full access to all data in the study and took responsibility for the integrity of the data and the accuracy of the data analyses.
